# Distal Colon Motor Dysfunction in Mice with Chronic Kidney Disease: Putative Role of Uremic Toxins

**DOI:** 10.3390/toxins10050204

**Published:** 2018-05-16

**Authors:** Elsa Hoibian, Nans Florens, Laetitia Koppe, Hubert Vidal, Christophe O. Soulage

**Affiliations:** 1 Univ. Lyon, CarMeN lab, INSERM U1060, INRA U1397, INSA de Lyon, Université Claude Bernard Lyon 1, F-69621 Villeurbanne, France; elhoibian@gmail.com (E.H.); nans.florens@chu-lyon.fr (N.F.); laetitia.koppe@chu-lyon.fr (L.K.); hubert.vidal@univ-lyon1.fr (H.V.); 2 Department of Nephrology, Hospices Civils de Lyon, Hôpital Edouard Herriot, F-69437 Lyon, France; 3 Department of Nephrology, Hospices Civils de Lyon, Centre Hospitalier Lyon-Sud, F-69495 Pierre-Bénite, France

**Keywords:** uremia, chronic kidney disease, gastrointestinal motility, colon, uremic toxins

## Abstract

Although gastrointestinal complications are a common feature of patients with chronic kidney disease (CKD), the impact of uremia on bowel motility remains poorly understood. The present study was, therefore, designed to investigate the impact of uremia on gut motility. Kidney failure was induced in mice by chemical nephrectomy using an adenine diet (0.25% *w/w*). Gastrointestinal transit time and colon motility were explored *in vivo* and *ex vivo*. Colons from control mice were incubated with uremic plasma or uremic toxins (urea, indoxyl-sulfate or p-cresyl-sulfate) at concentrations encountered in patients with end-stage renal disease. Mice fed an adenine diet for 3 weeks exhibited a 3-fold increase in plasma urea (*p* < 0.001) evidencing kidney failure. The median gastrointestinal transit time was doubled (1.8-fold, *p* < 0.001) while a reduction in colonic propulsive motility was observed in CKD mice (3-fold, *p* < 0.001). Colon from CKD mice exhibited an abnormal pattern of contraction associated with a blunted maximal force of contraction. Control colons incubated with plasma from hemodialysis patients exhibited a blunted level of maximal contraction (*p* < 0.01). Incubation with urea did not elicit any difference but incubation with indoxyl-sulfate or p-cresyl-sulfate decreased the maximal force of contraction (−66% and −55%, respectively. *p* < 0.01). Taken together, these data suggest that uremia impairs colon motility probably through the retention of uremic toxins. Colon dysmotility might contribute to the gastrointestinal symptoms often reported in patients with CKD.

## 1. Introduction

Gastrointestinal disorders are a common feature of patients with chronic kidney disease (CKD) [1]. Among the gastrointestinal complaints of CKD patients, dyspepsia, nausea, vomiting, abdominal pain, constipation and diarrheas are most commonly reported. However, the impact of an altered gut motility in CKD remains unclear. Some dysfunctions of the oesophagus [2] and stomach motility [3,4,5] have already been reported in patients with CKD. The cause–effect relationship between potential lesions and gut dysfunction is, nonetheless, ambiguous. Indeed, gastrointestinal symptoms occur in 40% of hemodialysis patients without detectable anatomical/histological lesions, while 60% of patients with evidenced gastric or duodenal lesions did not exhibit any gastrointestinal symptoms [6]. Intestinal motility disorders have also been reported in patients with end-stage renal disease paradoxally including diarrheas and constipation. On the one hand, disturbed small intestinal motility was found to be involved in diarrheas in some patients with CKD [7]. On the other hand, the prolongation of colonic transit time [8] was reported to contribute to constipation in hemodialysis patients. End-stage renal disease patients (ESRD, before initiation of renal suppletion therapy) exhibit normal or delayed transit time [9] and altered gastric myoelectrical activity [3]. Abnormal intestinal motility could further contribute to the intestinal malabsorption and thus to malnutrition/wasting observed in ESRD patients [10]. Experimental studies were performed to address the effects of renal dysfunction on gut motility. In a rat model of acute kidney injury (induced with gentamycin), a significant decrease in duodenum and colon motility was observed [11]. Lefèvre et al. demonstrated that duodeno-jejunal motility and colonic transit time were altered in dogs with moderate renal failure [12]. Rats with CKD (induced surgically by 5/6 subtotal nephrectomy) exhibited alteration of the interdigestive migrating complex (IMC), reduced gastric emptying and small intestinal transit [13,14]. However, limited data is available regarding the mechanisms that underlie gut dysmotility associated with renal failure. Several factors associated with uremia could account for the gastrointestinal tract dysmotility: (i) The level of several gastrointestinal hormones involved in gut motility (e.g., gastrin, cholecystokinin, motilin) are increased in CKD patients [15,16,17,18,19]; (ii) patients with CKD often exhibit autonomic dysfunction [20]; (iii) many compounds that accumulate in kidney failure may impair the contractility of gut smooth muscle. The present study investigated in a mouse model of CKD (induced non-surgically by chemical nephrectomy) the effect of renal failure on total gastrointestinal transit and mechanical activity of the gut. We further studied in vitro the impact of uremic plasma and some major uremic toxins on colon motility.

## 2. Results

### 2.1. Mice Fed an Adenin Diet Developed Renal Failure

The cumulative food intake was 275 ± 13 and 260 ± 21 kJ/mouse for control and CKD mice, respectively (*p* = 0.765). Biological data for each group are shown in Table 1. CKD mice exhibited a lower body weight than control mice (−23%, *p* < 0.001). Chemical nephrectomy resulted in a significant decrease in kidney mass (−39%, *p* < 0.001) and a cardiomegaly (heart weight +19%, *p* < 0.05). A macroscopic view of kidneys from control and CKD mice is shown as Supplementary Figure S1. CKD also exhibited a significant decrease in intestine weight while liver weights and colon weights were unaffected. CKD mice exhibited an elevated plasma urea (3.2 folds, *p* < 0.001) unambiguously evidencing the development of renal failure and a non-significant increase in proteinuria (*p* = 0.100).

### 2.2. Effect of Uremia on Gastrointestinal Transit Time

**Figure 1:**
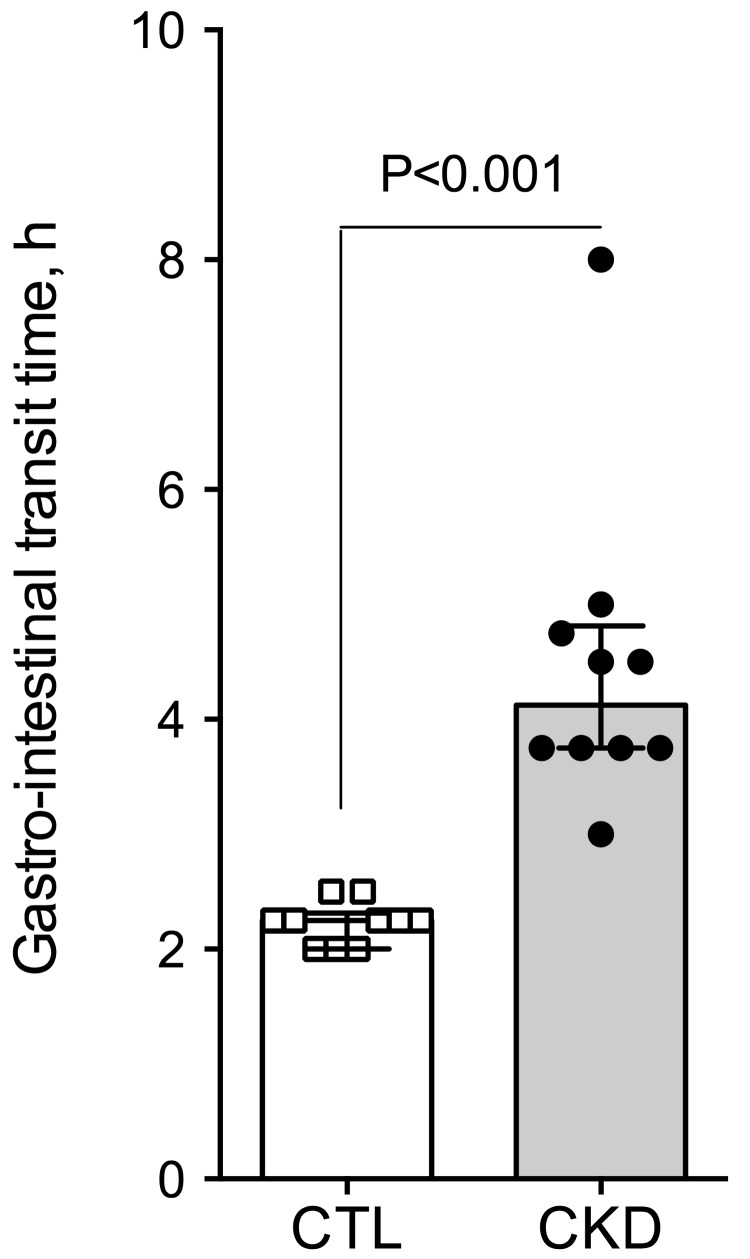
Alteration in gastro-intestinal transit time in mice with CKD. Whole gut transit time was measured by the carmine red method in control and CKD mice. Data are presented as median (interquartile range (IQR)) for *n* = 10 animals in each group. Data were compared using the Mann and Whitney U test. Differences were considered significant at the *p* < 0.05 level.

**Figure 2:**
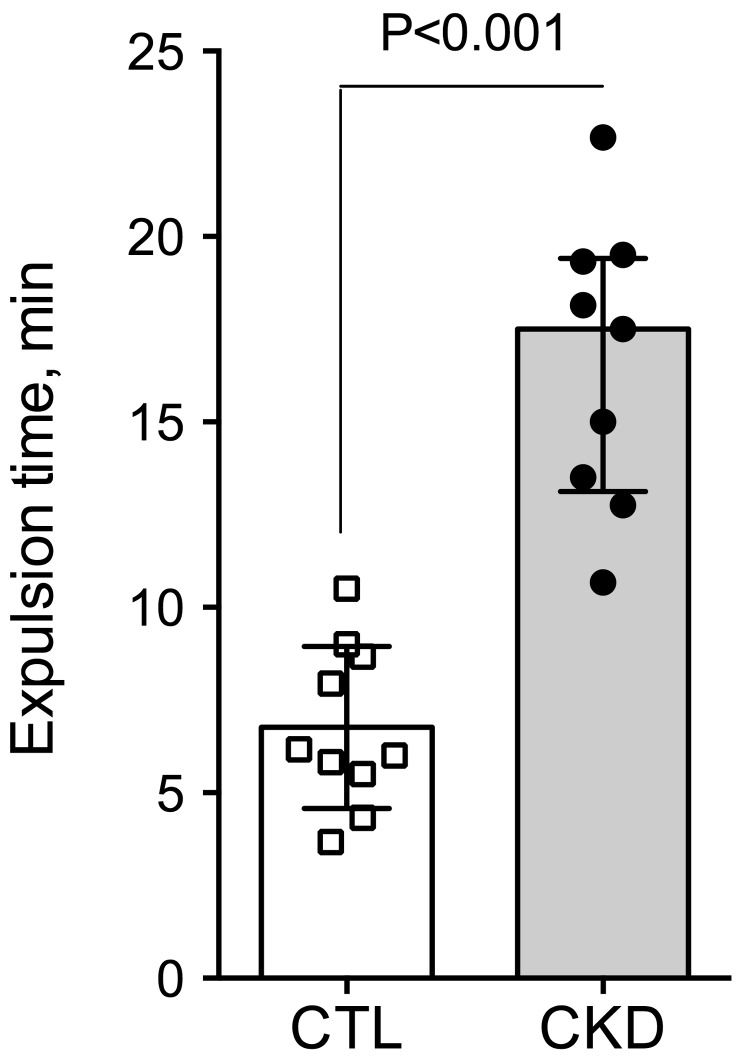
Reduced propulsive colonic motility in CKD mice. Distal colon motility was measured in vivo as the expulsion time for a 3 mm plastic bead inserted into the rectum. CKD mice exhibited a increased expulsion time evidencing a reduced colonic motility. Data are presented as median (IQR) for *n* = 10 animals in each group. Data were compared using the Mann and Whitney U test. Differences were considered significant at the *p* < 0.05 level.

**Figure 3:**
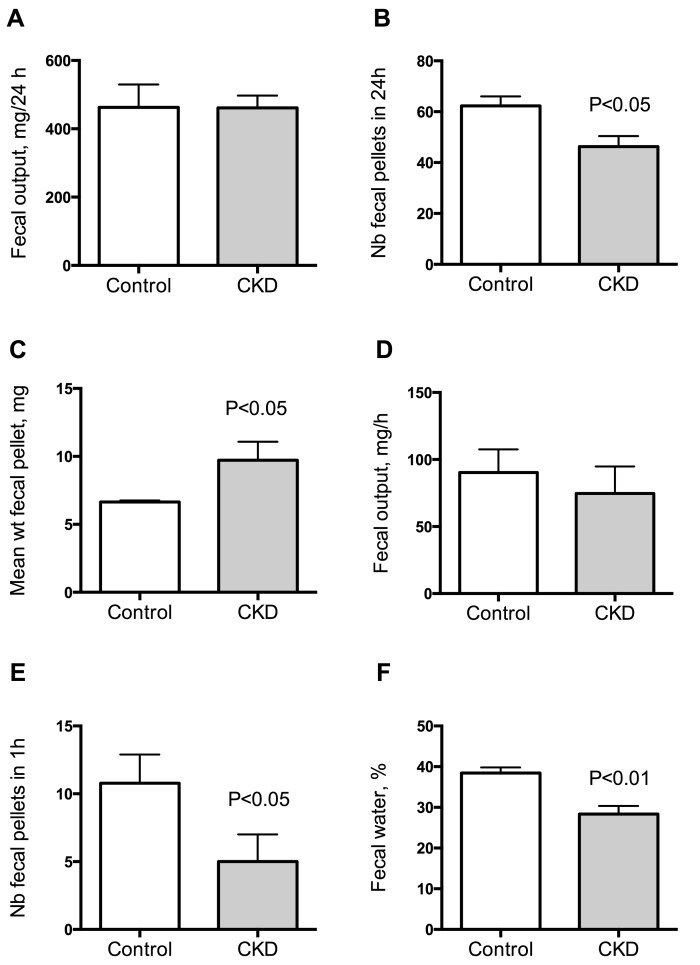
Measurement of fecal output in control and CKD mice. Fecal pellet output was measured over a 24 h period for 3 consecutive days in each mouse. The 3 day average for each mouse was used as a measurement of fecal output for that animal. Fecal output (**A**), number of fecal pellets (**B**) and mean weight of fecal pellets (**C**) in control and CKD mice. Fecal pellets were collected over a 1 h period for measurement of fecal water content. Wet weight (**D**), number of fecal pellets (**E**) and fecal water content (**F**) of feces collected over a 1 h period. Data are mean ± SD for *n* = 10 animals in each group. Differences were considered significant at the *p* < 0.05 level.

To investigate gut motility, gastrointestinal transit time, fecal pellet output and colon motility were measured *in vivo*. CKD mice exhibited an increase in total whole gut gastrointestinal transit time as estimated with carmine red (Figure 1). The median gastrointestinal transit time was 2.25 (interquartile range (IQR): 2.00–2.31) and 4.13 (IQR: 3.75–4.81) hours in control and CKD mice, respectively (2-folds increase, *p* < 0.001). A significant correlation was found between the plasma concentration of urea and the gastrointestinal transit time (Spearman r = 0.82; *p* < 0.001). A significant reduction of colonic motility in CKD mice was observed in the bead expulsion time assay (Figure 2). The expulsion time was 6.1 (IQR: 5.2–8.7) min and 17.5 (IQR: 13.1–19.4) min in control and CKD mice, respectively (*p* < 0.001). The fecal output was similar (Figure 3A) while the number of fecal pellets produced in 24 h (Figure 3B) was substantially lower in CKD compared to control mice. As a result, the mean weight of fecal pellets was higher in CKD mice (Figure 3C). Feces were collected over one hour to measure the fecal water content. CKD mice produced a reduced number of fecal pellets over the 1 h period while the fecal output was similar to control mice (Figure 3D,E). Fecal water content was significantly decreased in CKD mice (Figure 3F) supporting an increased colonic transit time as described in reference [12]. Taken together, these results suggested that uremia is associated with reduced propulsive colonic motility.

### 2.3. Effect of Uremia on Duodenum and Colon Motility

**Figure 4:**
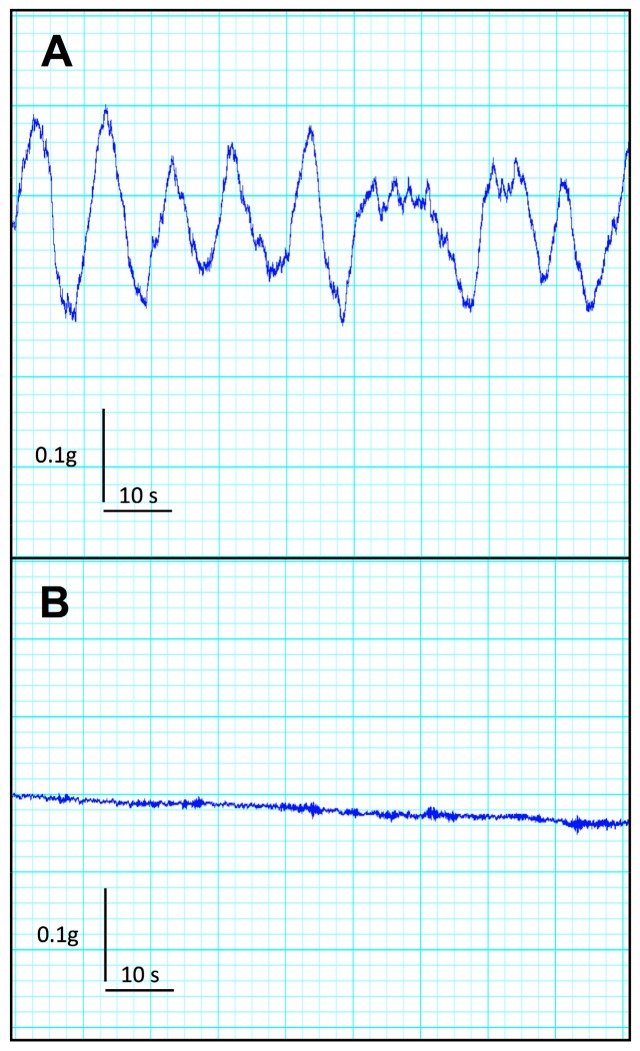
Typical mecanogram of isolated descending colon in control (**A**) and CKD mice (**B**). Note the absence of spontaneous motility in colon from CKD mice.

CKD mice exhibited no significant difference in duodenal frequency of contraction (*p* = 0.414), average duration of contraction (*p* = 0.355), average force of contraction (*p* = 0.414), or motility index (*p* = 0.288) as compared with the control group (see Table 2). In contrast, CKD mice exhibited a striking reduction in all descending colon motility parameters compared with the control group. Indeed, CKD mice exhibited a very low level of spontaneous activity and a blunted average force of contraction compared to control mice. A typical tracing of baseline descending colon motility in control and CKD mice is shown in Figure 4. The maximal contraction of a duodenum and colon segment was elicited using 1 mM potassium chloride, used as a depolarizing agent (Table 2). While no difference was noticed for the duodenum, the colon from CKD mice exhibited a sharp decrease in the maximal force of contraction (*p* < 0.001).

### 2.4. Uremic Plasma and Uremic Toxins Induce Gut Dysmotility

**Figure 5:**
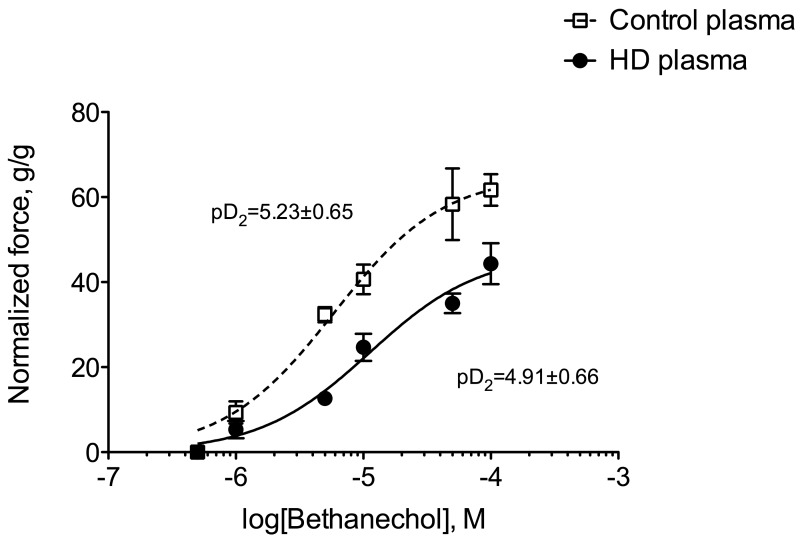
Comparison of in vitro cholinergic reactivity in the distal colon of control mice incubated with plasma from control or hemodialysis patients. Colons were incubated for 2 h with 20% (*v/v*) plasma from healthy volunteers or hemodialysis patients. Contraction of the smooth muscle of the colon was then elicited by stimulation with the muscarinic receptor agonist bethanechol (5.10^−7^–10^−4^ M). Data were expressed as mean ± SD for *n* = 4 independent experiments.

**Figure 6:**
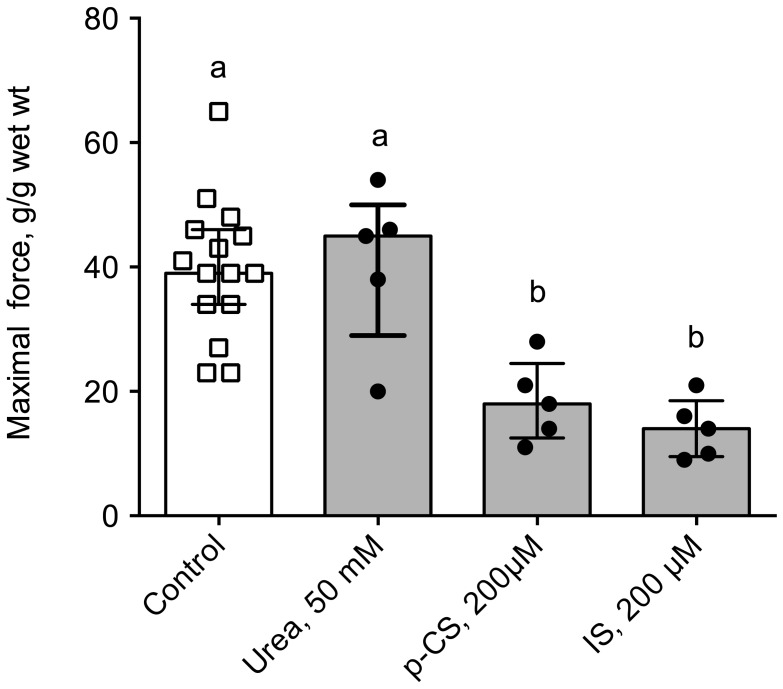
In vitro cholinergic reactivity in the distal colon of control mice incubated with uremic toxins. Colons were incubated for 2 h with urea (50 mM), p-cresyl sulfate (p-CS, 200 µM) or indoxyl-sulfate (IS, 200 µM). A maximal contraction of the smooth muscle of the colon was then elicited by stimulation with the muscarinic receptor agonist bethanechol (10^−4^ M). Data were expressed as median (IQR) for *n* = 5 independent experiments. Data were compared using the Krukall and Wallis test. Different letters indicate a significant difference between groups at *p* < 0.05 level.

Ex vivo, we used the muscarinic receptor cholinergic agonist bethanechol to elicit the contraction of a segment of distal colon of control mice incubated prior to the experiment with plasma from healthy volunteers or hemodialysis (HD) patients. The main demographic and clinical characteristics of healthy volunteers and HD subjects are shown in Table 3. Colon incubated with plasma from HD patients exhibited a blunted level of contraction when compared to those incubated with healthy plasma (Figure 5). Indeed, the maximal contraction was 30% lower in colon incubated with HD plasma compared to control (*p* < 0.001). In contrast, no difference was observed in cholinergic sensitivity as evidenced by the similar values of pD_2_ for bethanechol: 5.22 (95%CI: 5.06–5.51) and 4.91 (95%CI: 4.77–5.26) for control and CKD mice, respectively. When expressed as a percent of the maximal contraction, no difference was observed between the two conditions excluding any difference in sensitivity to the cholinergic agonist (data not shown). To get further insight into the uremic factors involved in colon dysfunction, we incubated colon from control mice with 3 major uremic toxins (i.e., urea, *p*-cresyl-sulfate and indoxyl-sulfate, see Figure 6) at the concentration encountered in patients with ESRD [21] according to the guidelines laid down by the European Workgroup on Uremic Toxins (EuTox, www.uremic-toxins.org) [22]. Incubation with urea (50 mM) did not elicit any difference but incubation for two hours with IS or p-CS sharply blunted the maximal contraction elicited by potassium chloride (−66% and −55%, respectively. *p* < 0.01).

## 3. Discussion

The gastro-intestinal symptoms in CKD patients are strongly related to gastrointestinal motor dysfunctions [4]. The aim of the present study was, therefore, to evaluate the effects of chronic kidney disease on intestinal motility in a mouse model of CKD. We further evaluated the impact of uremic plasma, collected from HD patients on the colon contractility. We observed that CKD mice exhibited an increased transit time and a blunted motility of the distal colon evidenced through *in vivo* as well as *ex vivo* experiments. We further observed that plasma from HD patients and some uremic toxins (i.e., IS and p-CS) impaired gut smooth muscle contractility.

The present results are in line with previous reports supporting a gut dysmotility in animal models or humans. Da Graça et al. reported an impaired gastric emptying in 5/6 nephrectomized rats [14]. Acute renal failure results decreased motility of the duodenum and the distal colon in rats [11]. In good agreement, Wu et al. reported that, total, right segmental, as well as rectosigmoïd segmental colonic transit times were prolonged in HD patients compared with healthy controls [8]. They further observed that increased individual colonic transit times were correlated with self-reported constipation in HD patients. In contrast, some other studies yielded some conflicting results. Dogs with moderate renal failure exhibit a decreased velocity in the myoelectrical migrating complex in the small intestine associated with a decreased transit time [12]. Fu et al. reported that the interdigestive myoelectric complex (IMC) of 5/6 nephrectomized rats was altered and based on fecal water content (used as a “proxy” of colonic transit time), and that the colonic transit time was decreased [13]. The different animal models (i.e., dogs, rats or mice) and the method used to produce the kidney failure (i.e., subtotal nephrectomy vs chemical nephrectomy) could account for such discrepancies between experimental studies. The level of kidney failure (i.e., moderate or terminal) reached in each experimental model could also account for these differences. The most common rodent model of CKD is the 5/6 subtotal nephrectomy model (also referred to as the “remnant kidney” model) in which there is a surgical reduction of the renal mass and nephrons number. Chemical nephrectomy using an adenine diet has, however, been proved to be a good model of renal failure [23]. The adenine diet induces a reproducible kidney failure [23,24] depending on the duration and the concentration of adenine in the diet [25]. The three weeks of adenine diet used in the present study produced irreversible kidney damage (see Table 1 and Supplementary Figure S1). The surgical process (i.e., 2-step nephrectomy) could per se have an impact on gastrointestinal transit (e.g., post-surgical adherences, fibrosis, ...). It is noteworthy that the use of a non-surgical model of CKD avoids these possible biases related to the surgical process. The study of gut motility (either *in vivo* or *in vitro*) was performed after a washout period of 3 to 4 weeks (i.e., mice were fed a normal chow for 3 or 4 weeks) excluding that adenine intake directly interfered with the measurement of motility. The adenine diet has already been successfully used to study the impact of renal failure on gut permeability [26,27]. Vaziri et al. [26] compared rats with renal failure induced either surgically by 5/6 subtotal nephrectomy or by chemical nephrectomy with adenine. Of note, no significant difference in gut structure or permeability was noticed between the two methods used to induce renal failure. In preliminary experiments, we incubated colon from control mice with a concentration of adenine 25 µM to mimic the adenine diet and failed to observe any difference of colon motility (data not shown). Thus, pre-incubation with adenine does not affect intestinal motility. The gut dysmotility is, therefore, an effect of kidney failure and uremia rather than an effect of adenine intake on the gut.

Only scarce data is available in the biomedical literature regarding the mechanisms that underlie impaired gut motility in kidney disease. Many factors could account for a reduced gut motility among which are: (i) abnormal levels of gastrointestinal hormones involved in the control of gut motility; (ii) autonomic nervous system dysfunction or imbalance; and (iii) the impact of the uremic environment. These putative mechanisms deserve some comments. Many gastrointestinal hormones display increased circulating levels in CKD (e.g., gastrin, motilin, cholecystokinin, vaso-intestinal peptide, …) [15,16,17,18,19,28,29,30]. Altered circulating levels of gastrointestinal hormones could have a strong impact on gut motility. Autonomic dysfunction in CKD leads to an increased sympathetic nervous system activity and depressed parasympathetic activity [20,31]. Since sympathetic system and parasympathetic systems, respectively, inhibit and stimulates gut motility, autonomic dysfunction could contribute to the dysmotility and the delayed gastrointestinal transit time observed in CKD. The fact that colon exhibits an abnormal motility *ex vivo*, however, challenges the role of autonomic dysfunction. An altered “humoral” environment including accumulation of inflammatory factors and/or uremic toxins could explain the colon dysfunction. This view is favoured by the significant negative correlation between plasma urea concentration and colon maximal force of contraction (r_s_ = 0.82; *p* < 0.001). To challenge this hypothesis, we incubated colon from control mice (i.e., healthy colons) with plasma from healthy volunteers or HD patients. Plasma from HD patients impaired the colon contraction elicited by a potent cholinergic agonist, suggesting that uremic plasma contains one or several factor(s) accumulated in CKD that could impair smooth muscle contraction. In good agreement, it was shown that HD treatment improves gastric hypo-motility and reduces the severity of gastrointestinal symptoms in CKD patients [32] suggesting that at least some of these factors could be removed by HD. Many compounds that accumulate in kidney failure may impair the contractility of gut smooth muscle among which many cytokines and uremic toxins. The “uremic syndrome” (also referred to as uremia) is indeed attributed to the progressive retention of numerous compounds, which in healthy individuals are normally excreted by the kidneys. The European Uremic Toxin Work Group (EuTox) listed about 90 uremic retention solutes (or uremic toxins) that accumulate in ESRD and exhibit deleterious effects on biological systems [21,22,33,34,35]. Based on their behaviour during dialysis process, uremic toxins were divided into three main groups: low-molecular-weight (MW) water-soluble compounds (<500 Da), large-molecular-weight compounds (the so-called “middle-molecules”, 0.5–60 kDa) and compounds tightly bound to plasma proteins (<500 Da). Of note, compounds belonging to the latter group are poorly removed by most of the conventional HD treatments due to their tight interactions with plasma proteins. Colon from control mice were incubated with some prototypical uremic toxins using relevant concentrations in the context of CKD [21,22]. Urea (60 Da) is the prototype of small water-soluble compounds. While older studies examining acute urea infusion suggested that urea only exhibited limited toxicity, more recent studies carefully re-evaluated the actual toxicity of urea [36]. In our hands, urea did not exhibit any effect on colon motility *ex vivo*. It was, however, demonstrated that urea, at a concentration relevant for uremia, triggers the disintegration of the gut epithelial barrier [37] by increasing gut permeability through the breakdown of the tight junctions. Furthermore, the effect of urea was dramatically amplified by the microbial enzyme urease added to simulate the presence of microbial flora [37]. Urease converts urea into ammonia, a compound shown to be highly toxic for the gut epithelia. However, we cannot rule out the role and the impact of ammonia on colon motility. Further experiments, directly using ammonia or urea in the presence of urease, are needed to confirm this point. IS (251 Da) and p-CS (188 Da) are prototypical of protein-bound uremic toxins. Colon incubated with IS or p-CS exhibited a blunted force of contraction (see Figure 6) suggesting that these toxins could have deleterious effect on colon smooth muscle cells. No data are available regarding the impact of p-CS/IS on visceral smooth muscle cells but extensive studies were conducted on vascular smooth muscle cells. p-Cresyl-sulfate was shown to damage vascular smooth muscle cells by inducing oxidative stress [38]. Indoxyl-sulfate was shown to promote proliferation [39,40] and calcification [41,42] of vascular smooth muscle cells, two hallmarks of the atherosclerotic process. Taken together, these data support the possible toxicity of these uremic toxins on visceral smooth muscle cells. These direct effects could explain the decrease in motility observed *in vivo* (e.g., reduced propulsive colon motility) but defects in proliferation or calcifications for instance cannot explain the effect observed *ex vivo* with incubation of control colon with uremic plasma or uremic toxins. However, as tissues were incubated with uremic toxins for two hours, oxidative stress or stimulation of cell-signaling pathways (such as for instance activation of aryl hydrocarbon receptor –AhR- by IS [43]) could account for the observed effects. We previously reported that incubation of muscle cells for 30 min with p-CS was sufficient to elicit biological effects [44]. Further studies are, however, needed to decipher the molecular mechanisms involved.

**Figure 7:**
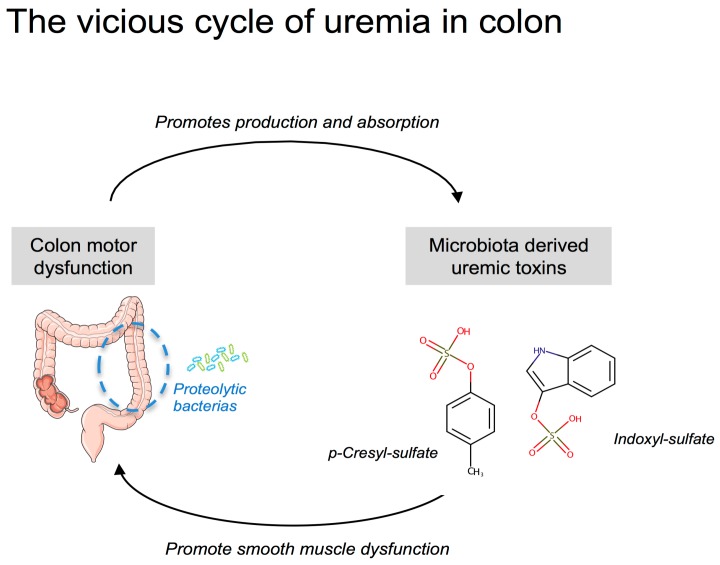
Illustration of the potential vicious cycle of uremia in the colon.

The putative consequences of an increased colonic transit time deserve some comments. The major role of the colon is to absorb water and electrolytes in order to concentrate the waste products of digestion in order to facilitate their disposal. The laxative bisacodyl was proved to be efficient to decrease serum potassium concentration in HD patients [45]. Thus, an increased residence time of stools in the colon could alter the absorption/secretion of potassium and contribute to the hyperkalaemia observed in CKD patients. Since the colon is a major site of water reabsorption, prolonged colon transit time could favour the formation of hard stools and promote constipation in CKD patients. The colon also provides an important reservoir for a microbial community, often referred to as the gut microbiota (thought to constitute 10^11^–10^12^ microbial cells per g of content). The colon is, nowadays, recognized as a major source of uremic toxins in CKD [46,47,48]. p-Cresyl-sulfate and IS are actually prototypes of uremic toxins originating from colonic bacterial metabolism. Pinpointing the major role of the gut microbiota, the survival time of bilaterally nephrectomized germ-free rats is doubled compared to that of conventional animals [49]. Colonic microbial metabolism can be categorized roughly as saccharolytic (i.e., carbohydrate fermentation) or proteolytic (i.e., protein fermentation). Carbohydrate fermentation predominates in the right (i.e., ascending) colon while protein fermentation predominates in the left (i.e., descending) colon [50,51]. Saccharolytic metabolism is considered beneficial for the host while proteolytic metabolism generates many toxic by-products such as p-cresol and indole (i.e., precursors of pCS and IS, respectively). Increasing the residence time in the right colon could, therefore, promote the production and the absorption of these uremic toxins [52]. Interestingly, Cummings et al. reported a significant correlation between a longer colonic transit time and urinary excretion of phenols [53]. In good agreement, treatment of CKD mice with lubiprostone (a drug commonly used for the treatment of constipation) decreased the plasma level of uremic toxins derived from gut microbiota, such as indoxyl sulfate and hippuric acid [27]. Such a phenomenon could, therefore, promote a vicious cycle between the production of colon-derived uremic toxins and their direct impact on colon motility (see scheme in Figure 7).

## 4. Conclusions

In conclusion, this study might confirm that the colic motility is altered in CKD, as evidenced by an increased gastrointestinal transit time and a reduced propulsive colonic motility. The gastro-intestinal complaints in CKD patients seem to be related to gastrointestinal motor dysfunction [4]. We speculate that reduced colon propulsive motility and increased colonic transit time could strongly contribute to the constipation often observed in patients with CKD. The increased residence time of the stools in the colon might further increase the production/absorption of colon-derived uremic toxins and alter the reabsorption of water and electrolytes. As previously mentioned by Poesen et al. [51], colon motility could be an emerging target for therapeutic interventions in uremic patients.

## 5. Materials and Methods 

### 5.1. Chemicals and Reagents

Unless otherwise stated, all chemicals were obtained from Sigma Aldrich (Saint Quentin Fallavier, France). Indoxyl sulfate (IS, ref I3875) was purchased from Sigma Aldrich (Saint Quentin Fallavier, France). The potassium salts of *p*-cresyl sulfate (*p*-CS) was synthesized according the method described by Feigenbaum and Neuberg [54]. Since *p*-CS and IS are mainly protein-bound in biological systems, all in vitro experiments were performed in a medium supplemented with 35 g/L bovine serum albumin (BSA) according to the recommendations of EuTox [22]. Since p-CS and IS were synthesized (or purchased) as potassium salts, a final concentration of 200 µM KCl in saline was chosen as control to equal the potassium concentration in the K-salt of *p*-CS or IS.

### 5.2. Patients

Nineteen hemodialysis patients and 11 non-CKD controls were recruited from an ongoing study at Hospices Civils de Lyon (Department of Nephrology). The patient characteristics are presented in Table 3. The study was conducted in accordance with the Declaration of Helsinki and was approved by the local ethical committee (reference L16-57 and DC-2009-1066, CPP Lyon Sud-Est IV). Written informed consent was obtained from all subjects. Blood samples were collected, centrifuged at 1500× *g* for 10 min to isolate plasma. Plasma from non-CKD and hemodialysis (HD) patients were pooled in equal amounts and thereafter referred to as ‘Control pool’ and ‘HD pool’, respectively. The control pool and HD pool were sterile filtered (cut-off 0.22 µm), aliquoted, and stored at −80 °C until use.

### 5.3. Animals

Animal experiments were performed under the authorization n°69-266-0501 (INSA-Lyon, DDPP-DSV), according to the guidelines laid down by European Union Council Directive 2010/63UE. The project was approved by the local institutional review board for animal care and use (CETIL) under the reference # 3210 (29 February 2016). Three-week old male C57Bl/6 JRj mice were purchased from Janvier SA (Le Genest-Saint-Isle, France) and housed in an air-conditioned room with a controlled environment of 21 ± 0.5 °C and 60–70% humidity, under a 12 h light/dark cycle (light on from 07:00 to 19:00) with free access to standard food (A04, SAFE, Augy, France) and water. CKD was induced non-surgically by feeding the mice with an adenine-enriched diet. Thirty mice were randomized to either a CKD (*n* = 15) or a control group (*n* = 15). The animals assigned to the CKD group were fed a chow containing 0.25% (*w/w*) adenine on A04 basis (SAFE, Augy, France) for 21 days. The control animals were pair-fed with a regular chow (A04) throughout the period of induction of CKD. Then, all mice were fed with standard diet for 21 days prior to the experiments. 

### 5.4. Measurement of Total Gastrointestinal Transit Time

Carmine red (Sigma-Aldrich, Saint Quentin Fallavier, France) was prepared as a 6% (*w/v*) solution in 0.5% methyl cellulose and sterile-filtered. Mice were individually housed and fasted overnight. Mice were force-fed with 0.3 mL of the carmine red solution between 09:00 and 10:00 AM and monitored every 30 min until the appearance of the first red fecal pellet. The time from gavage to the appearance of the first red pellet was recorded as total gastrointestinal transit time [55].

### 5.5. Urine Collection and Biochemical Measurements

The mice were individually placed into metabolic cages to collect 24-hour urine. Urine volume was determined gravimetrically and urine protein concentration was measured with Bradford protein assay (BioRad, Marne-la-Coquette, France) using BSA as standard. The plasma concentration of urea was determined using an enzymatic kit from Sobioda (Montbonnot, France). 

### 5.6. Fecal Water Content

Fecal pellet output was evaluated in control and CKD mice. Mice were individually housed in a metabolic cage for 3 consecutive days with food and water available ad libitum. Fecal pellets were collected daily from each mouse and pellets were counted and weighed to the nearest milligram. Fecal pellets were placed into a 60 °C oven overnight and dry weight was then determined. Fecal pellet wet and dry weights from each animal was averaged over the 3-day period and that average value was used for each mouse.

### 5.7. Measurement of Colonic Motility In Vivo

Colonic motility was assessed in vivo by measuring time to expulsion of a 3 mm diameter plastic bead inserted into the descending colon. Mice were fasted 12 h prior to experimentation. A single plastic bead was inserted 15 mm into the distal colon of control and CKD mice and expulsion time was measured [56].

### 5.8. Sacrifice and Tissue Dissection

Animals were deeply anesthetized with sodium pentobarbital (100 mg/kg i.p.). Blood was collected through cardiac puncture in a heparinized syringe. Blood was centrifuged for 5 min at 3500× *g* to obtain plasma. Plasma were snap frozen in liquid nitrogen and stored at −80 °C until analysis. A part of the duodenum and of the descending colon were rapidly dissected out and ice-cold saline for ex vivo experiments as described below. Liver, heart, kidneys, small intestine, colon were dissected out, weighed, snap frozen in liquid nitrogen and stored at −80 °C. 

### 5.9. Isolated Duodenum and Colon

Segments of either duodenum or descending colon (approximately 0.5 cm long) were mounted onto stainless steel supports and suspended in an organ tissue bath containing 20 mL of warmed (37 °C) Tyrode’s solution (containing in mM: 137 NaCl, 2.7 KCl, 0.9 CaCl_2_, 0.5 MgCl_2_, 0.4 NaH_2_PO_4_, 11.9 NaHCO_3_, and 5.5 D-glucose, adjusted to pH 7.40) continuously bubbled with air. The tissues were connected to an isometric force transducer (AD Instrument, Paris, France), connected to an amplifier (Powerlab 10T, AD Instrument, Paris, France) and a computerized acquisition system (Chart 5.3, AD instrument, Paris, France) to record changes in isometric force. The resting tension was adjusted to 1 g and the tissues were then equilibrated for 30 min. Baseline motility was recorded for 30 min. Then, a record was obtained in the presence of 1 mM of potassium chloride (KCl). For evaluation of basal intestinal motility, the frequency of contraction, average duration of contraction, average force of contraction, and motility index was computed from the record. Duodenum and colon motility was expressed as the motility index calculated as Ln (number of peaks × sum of peak amplitudes +1) [57]. The absolute force of contraction in response to KCl was measured. At the end of the experiments, the segment of duodenum or colon used for the recording was blotted dry and weighed to the nearest milligram for normalization of tension (expressed as g/g of wet weight). In a second set of experiments, mice were euthanized by inhalation of carbon dioxide according to the American Veterinary Medical Association (AVMA) guidelines and as approved by the regional ethics committee (CETIL, CNREEA n°102). Tissues were incubated with 20% (*v/v*) of plasma from the Control or HD pools. The muscarinic cholinergic receptor agonist, bethanechol (5.10^−7^ to 10^−4^ M) was then tested to determine visceral smooth muscle reactivity in the colon. Some tissue was also incubated for 2 h with urea (50 mM), *p*-CS (200 µM) or IS (200 µM). Bethanechol (10^−4^ M) was then used to elicit the maximal contraction of the colon segment. At the end of the experiments, the section of colon used for the recordings were blotted dry and weighed to the nearest milligram for normalization of tension (expressed as g/g of wet weight). pD_2_ was calculated as –log_10_(EC_50_) with EC_50_, effective concentration 50% of bethanechol.

### 5.10. Statistics

Data are expressed as means ± standard deviation (SD) or median (interquartile range) and were analyzed using the GraphPad Prism, version 5.0, software (GraphPad software, La Jolla, CA, USA). Simple comparisons were performed using Mann and Whitney U tests. Multiple comparisons were performed using Kruskall and Wallis tests and appropriated with Dunn’s tests. Differences were considered significant at the *p* < 0.05.

## Figures and Tables

**Table 1:** Biometry and biochemistry in control and chronic kidney disease (CKD) mice.

	Control (*n* = 16)			CKD (*n* = 15)			*p*-Value
**Biometry**							
Body weight, g	20.6	±	0.2	15.9	±	0.8	<0.001
Kidney, mg/10 g bw	126	±	9	77	±	12	<0.001
Heart, mg/10 g bw	53	±	7	63	±	11	0.023
Liver, mg/10 g bw	408	±	13	415	±	50	0.529
Intestine, mg/10 g bw	335	±	20	385	±	35	<0.001
Colon, 10 mg bw	53	±	9	65	±	14	0.065
**Biochemistry**							
Plasma urea, mmol/L	8.6	±	0.8	27.9	±	4.0	<0.001
Proteinuria, mg/24 h	7.9	±	6.9	36.0	±	21.8	0.100

Data are mean ± standard deviation (SD). Abbreviations: bw, body weight. Differences were considered significant at the p < 0.05 level.

**Table 2:** Duodenum and colon motility parameters in the control and CKD mice.

	Control (*n* = 6)			CKD (*n* = 5)			*p*-Value
**Duodenum**							
Frequency of contraction, min^−1^	36.3	±	4.5	30.7	±	4.7	0.583
Average duration of contraction, s	1.8	±	0.3	2.3	±	0.4	0.355
Average force of contraction, g/g wet wt	2.4	±	0.6	1.8	±	0.6	0.485
Maximal force of contraction, g/g wet wt *	6.6	±	3.3	6.5	±	1.4	0.920
Motility index, AU	8.8	±	0.4	7.0	±	1.5	0.288
**Descending colon**							
Frequency of contraction, min^−1^	6.8	±	3.5	<1			-
Average duration of contraction, s	14.1	±	6.7	nd			-
Average force of contraction, g/g wet wt	3.7	±	1.4	0.07	±	0.03	0.016
Maximal force of contraction, g/g wet wt *	35.5	±	11.2	0.9	±	0.6	<0.001
Motility index, AU	8.8	±	1.1		nd		-

Data are mean ± SD. AU, arbitrary unit. * Maximal force of contraction was elicited with 1 mmol/L of KCl.

**Table 3:** Demographic and clinical characteristics of the subjects.

	Control (*n* = 11)			Hemodialysis (*n* = 19)			*p*-Value
Gender, M/F	6/5			13/6			0.696
Age, y	49.0	±	12.9	62.4	±	14.6	0.017
Dialysis vintage, y		n/a		3.7	±	2.6	-
Creatinine, µM	80	±	32	818	±	402	<0.001
eGFR, mL/min.1.73m^2^	94	±	29	<15			-
Urea, mM	6.6	±	2.7	20.7	±	8.0	0.007

Data are expressed as means ± SD. Means were compared with Student t-test. Proportions were compared using Fisher’s exact test. Statistical significance was set at the *p* < 0.05 level. Abbreviations: CKD, chronic kidney disease; eGFR, estimated glomerular filtration rate; n/a, not applicable. GFR was estimated according to the CKD-EPI formula.

## Supplementary Materials

The following are available online at http://www.mdpi.com/2072-6651/10/5/204/s1. Figure S1: Macroscopic view of the kidney of control and CKD mice.
