# Supplementary material for: Distal Colon Motor Dysfunction in Mice with Chronic Kidney Disease: Putative Role of Uremic Toxins
Source: Toxins (Basel). 2018 May 16;10(5):204. doi: 10.3390/toxins10050204 (PMC5983260; doi:10.3390/toxins10050204)
Supplement: Supplementary file 1 [file toxins-10-00204-s001.pdf]

Supplementary Materials: Distal Colon Motor Dysfunction in Mice with Chronic Kidney Disease: Putative Role of Uremic Toxins

Elsa Hoibian, Nans Florens, Laetitia Koppe, Hubert Vidal and Christophe O. Soulage

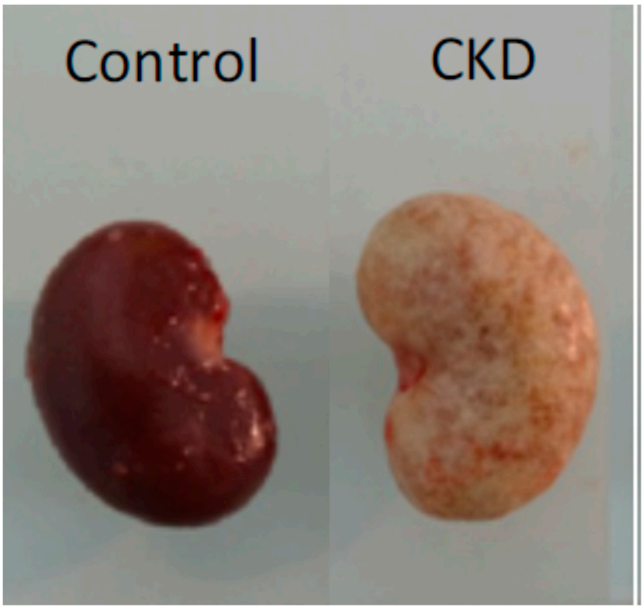

Figure S1. Macroscopic view of the kidney of control and CKD mice. Mice were fed for 3 weeks with a diet containing 0.25% (w/w) adenin to induce kidney failure. Note the fibrotic aspect of the kidney from CKD mice.
